# Characteristics of Patients with De Novo Metastatic Breast Cancer and Positive Human Epidermal Growth Factor Receptor 2 in a Reference Center in Mexico

**DOI:** 10.7759/cureus.100994

**Published:** 2026-01-07

**Authors:** Rogelio J Martinez-Samano, Mireya López-Gamboa

**Affiliations:** 1 School of Medicine, Universidad Nacional Autonoma de Mexico, Mexico City, MEX; 2 Pharmacovigilance Institutional Center, Instituto Nacional de Cancerología, Mexico City, MEX

**Keywords:** breast cancer, descriptive study, her2-positive, metastases, retrospective studies

## Abstract

Background

Breast cancer is an important disease with a high burden worldwide; approximately 5% to 10% of cases are diagnosed as metastatic breast cancer. A subset of patients with human epidermal growth factor receptor 2 (HER2) positivity and stage IV disease at diagnosis are identified as having HER2-positive de novo metastatic breast cancer, which is poorly characterized.

Methods

A retrospective cohort study was performed at the National Cancer Institute in Mexico City using 10 years of collected data. Variables included patient status, sociodemographics, and clinical, pathological, treatment patterns, and receptor status, while overall survival (OS) and progression-free survival (PFS) were also estimated. For assessing possible differences between receptor-status groups, a Fisher's exact test was performed. Survival analyses were performed using Kaplan-Meier plots, and a log-rank test was used to evaluate possible differences. Multivariate survival analyses for social and demographic variables were performed using Cox’s proportional hazard model.

Results

A total of 145 patients were identified, and out of them, 40.7% were classified as HER2+ ER+ PR+, 40% as HER2+ ER- PR-, 13.1% as HER2+ ER+ PR-, and 6.2% as HER2+ ER- PR+. The median OS of all patients was 96 months (95% confidence interval (CI): 79 to not estimable) and PFS was 18.7 months (95% CI: 14.5-27.3). Survival rates for all patients were 94%, 84%, 77%, 73%, and 66% for years one through five, respectively. Only few statistical differences were found in descriptive analysis; in the log-rank test and Cox’s proportional hazard model, no differences or associations were found.

Conclusions

Breast cancer is a leading cause of mortality and morbidity worldwide with several different outcomes depending on the clinical stage and molecular profile. Recently, de novo metastatic breast cancer has been studied because of positive outcomes after treatment, and several therapeutic strategies have been employed to increase patients’ survival rate. This study provided descriptive results of clinical, sociodemographic, treatment patterns, and pathological characteristics in HER2-positive de novo metastatic breast cancer in patients according to molecular receptor status. No relevant statistical significance was found; the exploratory survival outcomes should be interpreted as exploratory due to high lost-to-follow up rate. These results could be useful in terms of design of future clinical trials.

## Introduction

Breast cancer (BC) is one of the leading causes of mortality and morbidity worldwide; it is the most common cancer in women and the second leading cause of death among women [1], with an estimated 2.3 million cases and 685,000 deaths in 2020 [2]. BC represents approximately 24.5% of all cancers and 15.5% of cancer deaths; it ranks first in incidence and mortality in developing countries [2].

Among patients with BC, approximately 5%-10% are diagnosed with metastases at the initial physician visit. Those patients with BC who present with synchronous macroscopic sites of distant disease at diagnosis are considered to have de novo metastatic BC (dnMBC) [3]. A subset of dnMBC cases includes those that have HER2 (human epidermal growth factor receptor 2) enriched in tumor tissue; proportionally, HER2-positive BC occurs with more frequency than HER2-negative in de novo settings [3]. Also, the expression of hormonal receptor (HR) is associated with several outcomes, but its relevance in dnMBC is not fully described [4].

Survival in BC is influenced by race, geography, socioeconomic status, and insurance status, which complicates the effort to identify the determinants of survival in patients with dnMBC [5]. In HER2-positive and HR-positive dnMBC, targeted therapy is essential and constitutes the backbone of treatment. The efficacy and safety of these molecules has been extensively studied [6].

The aim of this retrospective research is to characterize the social, clinical, treatment patterns, and pathological characteristics of HER2-positive dnMBC in a cancer care reference center in Mexico, describing the possible differences according to the HR status. In addition, a survival analysis was performed as an exploratory way to infer possible associations between variables and outcomes.

## Materials and methods

This was a retrospective cohort study carried out in the National Cancer Institute in Mexico City with the participation of patients with dnMBC. To obtain records from target patients, a search of electronic health records was made based on a list provided by the statistics and archive department and selectively filtered after reviewing clinical information about cancer histories and the fulfillment of criteria for HER2-positive dnMBC. The time for data collection was 10 years (from January 2010 to December 2020). Data was collected prospectively and retrospectively outside this time window to ensure the collection of all relevant variables for survival analysis. The cut-off for analysis was May 2024.

The collected variables were patient status (alive, dead, and lost to follow-up); sociodemographics (age, marriage status, medical insurance status, geographical origin, level of education, and occupation); clinical data (age of menarche, menopausal status, births, breast cancer laterality, tumor, node, metastasis (TNM) status, and metastasis number and localization); treatment patterns (surgery, radiotherapy, lines of treatment) and pathological features (cancer type; pattern; Scarff-Bloom-Richardson (SBR) status; desmoplasia; presence of inflammatory cells; lymphatic, vascular and neural permeation; estrogens (ER), progesterone (PR), HER2 receptor status; and KI67 expression reported as a percentage of expression in the tumoral tissue). The receptor quantification was according to institutional procedures (Allred score) using internal cut-off thresholds for defining positivity. For assessing possible differences between groups, Fisher’s exact tests were performed to compare receptor-status groups for categorical data.

As an exploratory analysis, survival techniques were used to describe the possible effect of receptor status, and sociodemographics in survival. Overall survival (OS) was calculated from the first time the patient received any treatment (biological or chemotherapy) for BC until death, last consultation, or lost to follow-up (LFU); progression-free survival (PFS) was calculated from first time patient received any treatment until clinical or radiological disease progression (whichever happened first) according with medical records. Survival analyses were performed using Kaplan-Meier plots and missing or incomplete data were censored in the analysis of time-to-event endpoints. A log-rank test was used to evaluate possible differences between groups. After proportionality assumptions were tested, a multivariate survival analyses for social and demographic variables were performed with OS data using Cox’s proportional hazard model, and the results were reported as hazard ratios with 95% confidence intervals (CI). The protocol was assessed by the local Research and Ethics Committee and approved with the number SR01/CI/01-2020.

All statistical procedures were performed using R software, version 24.0 (R Foundation for Statistical Computing, Vienna, Austria). In the statistical test, p<0.05 was considered statistically significant.

## Results

Patients and status

**Figure 1:**
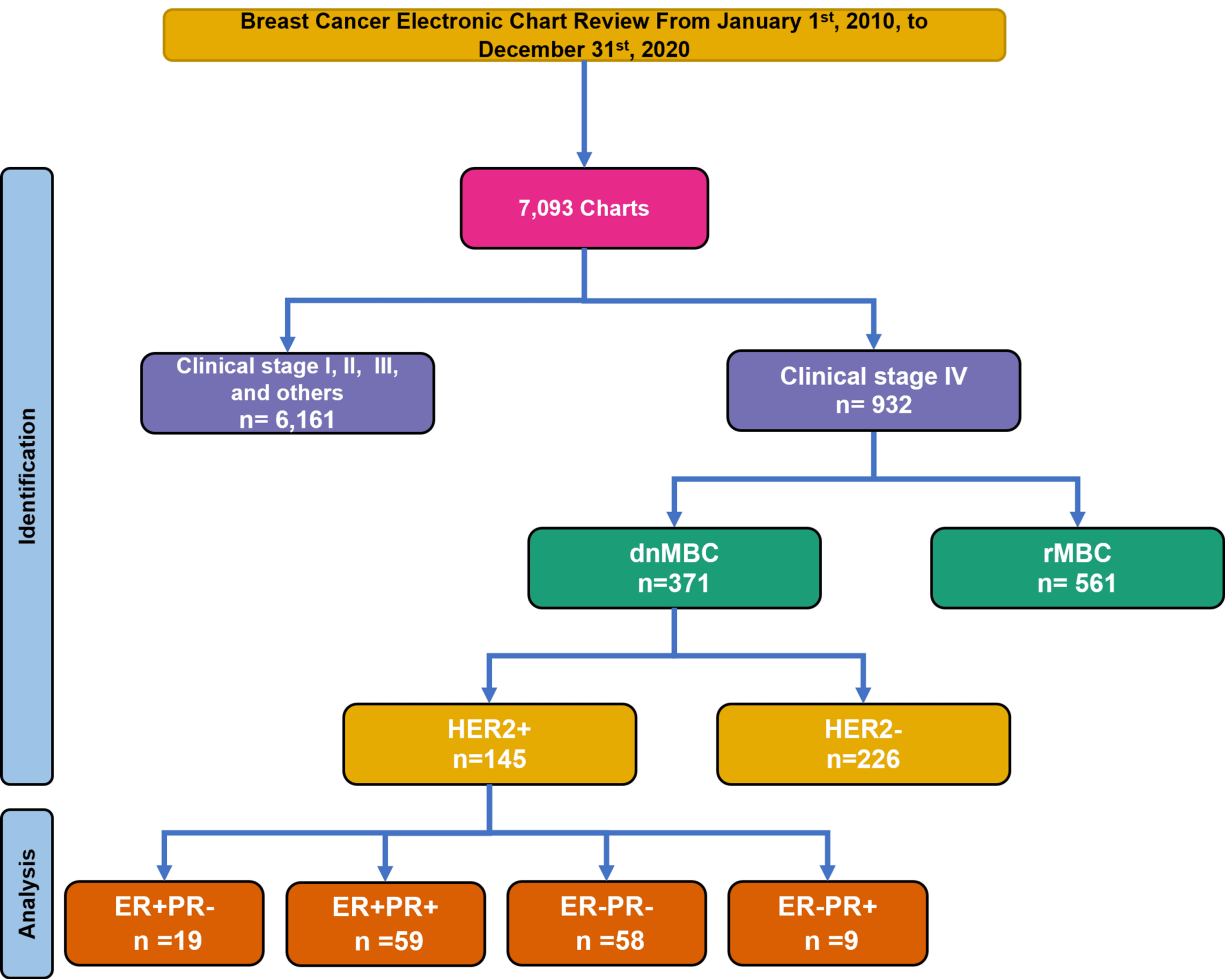
Flow chart for identifying patients with HER2-postive dnMBC according with hormonal receptors dnMBC=de novo metastatic breast cancer, rMBC=recurrent metastatic breast cancer, HER2=human epidermic growth factor, receptor 2; ER=estrogen receptor; and PR=progesterone receptor.

Of the 7,093 records retrieved of patients with BC, only 145 met the criteria for HER2-positive dnMBC. According to histopathological and immunohistochemical results, 59 patients (40.7%) were stratified as HER2+ER+PR+, 58 (40%) as HER2+ER-PR-, 19 (13.1% ) as HER2+ER+PR-, and nine (6.2%) as HER2+ER-PR+ (Figure 1). At the time of analysis (May 2024), 49 patients (33.8%) were still alive, 42 (29%) had died, and 54 (37.2%) were lost to follow-up (Table 1). The total follow-up time was (median, range) 42 months (1-166 months) for all patients; 39 months (1.4-10 months) for the HER2+ER+PR- group, 44 months (3.3-121 months) for the HER2+ER+PR+ group, 35 months (27 days to 108 months) for the HER2+ER-PR- group, and 30 months (11.5-166 months) for the HER2+ER-PR+ group.

**Table 1:** Status of dnMBC patients according to ER and PR expression Values are presented as number (n) and frequency (%). dnMBC=De novo metastatic breast cancer; LFU=lost to follow-up; HER2=human epidermic growth factor, receptor 2; ER=estrogen receptor; PR=progesterone receptor.

Status	All Patients (n, %)	HER2+ER+PR- (n, %)	HER2+ER+PR+ (n, %)	HER2+ER-PR- (n, %)	HER2+ER-PR+ (n, %)
Alive	49 (33.8)	6 (4.1)	20 (13.8)	20 (13.8)	3 (2.1)
Dead	42 (29)	8 (5.5)	14 (9.7)	18 (12.4)	2 (1.4)
LFU	54 (37.2)	5 (3.4)	25 (17.2)	20 (13.8)	4 (2.8)
Total	145 (100)	19 (13.1)	59 (40.7)	58 (40)	9 (6.2)

Sociodemographics

Sociodemographic characteristics are shown in Table 2. All patients were women aged 52 years (28-89 years) (median, range), split as 52.1 years (29-72 years) in the HER2+ER+PR- group, 53.7 years (34-89 years) in the HER2+ER+PR+ group, 50.8 years (31-67 years) in the HER2+ER-PR- group and 48.1 years (28-62 years) in the HER2+ER-PR+ groups. Out of the participants, 61 (42.1%) were married, 38 (26.2%) were single, 18 (12.4%) were widowed, 16 (11%) practiced common-law marriage and 12 (8.3%) were divorced. One hundred and thirty-seven patients (94.5%) did not have any insurance, whereas eight (5.5%) patients had social-medical insurance. As regards the geographical origin, 124 patients (85.5%) came from the center of Mexico, 19 (13.1%) from the southeast and two (1.4%) from west of the country. No statistical differences were found for any variable or group.

**Table 2:** Sociodemographic characteristics of dnMBC patients according to ER and PR receptor expression Values are presented as number (n) and frequency (%). HER2=Human epidermic growth factor, receptor 2; ER=estrogen receptor; and PR=progesterone receptor.

Characteristics	All Patients (n, %)	HER2+ER+PR- (n, %)	HER2+RE+RP+ (n, %)	HER2+RE-RP- (n, %)	HER2+RE-RP+ (n, %)
Marital status					
Married	61 (42.1)	6 (4.1)	26 (17.9)	27 (18.6)	2 (1.4)
Divorced	12 (8.3)	1 (0.7)	2 (1.4)	6 (4.1)	3 (2.1)
Single	38 (26.2)	7 (4.8)	16 (11)	13 (9)	2 (1.4)
Common-law	16 (11)	1 (0.7)	7 (4.8)	6 (4.1)	2 (1.4)
Widowed	18 (12.4)	4 (2.8)	8 (5.5)	6 (4.1)	0 (0)
Insurance status					
Insured	8 (5.5)	0 (0)	4 (2.8)	4 (2.8)	0 (0)
Not insured	137 (94.5)	19 (13.1)	55 (37.2)	54 (37.2)	9 (6.2)
Geographical origin					
Center	124 (85.5)	15 (10.3)	51 (35.2)	51 (35.2)	7 (4.8)
Southeast	19 (13.1)	4 (2.8)	6 (4.1)	7 (4.8)	2 (1.4)
West	2 (1.4)	0 (0)	2 (1.4)	0 (0)	0 (0)
Educational level					
Illiterate	9 (6.2)	2 (1.4)	3 (2.1)	4 (2.8)	0 (0)
Bachelor’s	15 (10.3)	3 (2.1)	8 (5.5)	4 (2.8)	0 (0)
High school	23 (15.9)	3 (2.1)	8 (5.5)	9 (6.2)	3 (2.1)
Elementary	54 (37.2)	5 (3.4)	21(14.5)	24 (16.6)	4 (2.8)
Secondary	40 (27.6)	6 (4.1)	18 (12.4)	15 (10.3)	1 (0.7)
Technical	4 (2.8)	0 (0)	1 (0.7)	2 (1.4)	1 (0.7)
Occupation					
Housewife	112 (77.2)	13 (9)	45 (31)	46 (31.7)	8 (5.5)
Unemployed	5 (3.4)	0 (0)	2 (1.4)	2 (1.4)	1 (0.7)
Retired	1 (0.7)	0 (0)	0 (0)	1 (0.7)	0 (0)
Non-professional	17 (11.7)	3 (2.1)	6 (4.1)	8 (5.5)	0 (0)
Professional	9 (6.2)	3 (2.1)	5 (3.4)	1 (0.7)	0 (0)
Technical	1 (0.7)	0 (0)	1 (0.7)	0 (0)	0 (0)

Conversely, the level of education was identified in all patients: 54 (37.2%) had elementary education, 40 (27.6%) secondary education, 23 (15.9%) high school education, and 15 (10.3%) a bachelor’s degree; nine (6.2%) were illiterate and four (2.8%) had technical training. Most patients were housewives (112 patients, 77.2%) followed by no professional activities (17 patients, 11.7%), professional activities (nine patients, 6.2%), unemployed (five patients, 3.4%), and retired and technical (one patient each, 0.7%). No statistical differences were found for any variable or group.

Clinical data

The age at menarche (average, range) was 12.9 years (8-18 years) for all patients, with 12.7 years (8-18 years) in the HER2+ER+PR- group, 13 years (9-18 years) in the HER2+ER+PR+ group, 12.8 years (9-16 years) in the HER2+ER-PR- group, and 13.8 years (13-15 years) in the HER2+ER-PR- group. According to medical records, five patients (3.4%) were classified as menopausal, 68 (46.9%) as premenopausal and 72 (49.7%) as postmenopausal. We also documented parity: 66 patients (45.5%) had zero to two deliveries, 61 (42.1%) had three to five deliveries, and 18 (12.4%) had more than five deliveries. No statistical differences were found for any variable or group.

Regarding the clinical characteristics of BC, 77 patients (53.1%) had a tumor in the right mammary gland, 62 (42.8%) in the left gland, and six (4.1%) were diagnosed with double synchronic BC (bilateral). Tumor burden was reported with T4 status in 90 patients (62.1%), followed by T3 in 25 patients (17.2%), T2 in 23 patients (15.9%), Tx in four patients (2.8%), and T1 in three patients (2.1%). Also, N3, N2, N1, N0, and Nx were documented in 93 (64.1%), 29 (20%), 19 (13.1%), three (2.1%) patients, and one (0.7%) patient, respectively. Metastatic disease was measured as the number of metastatic sites in increasing order: 68 (46.9%), 39 (26.9%), 23 (15.9%), eight (5.5%), three (2.1%), three (2.1%) patients and one (0.7%) patient had one, two, three, four, five, six, and seven sites of secondary disease. Finally, visceral metastatic disease was found in 75 patients (51.7%). Statistical differences were found only in metastasis sites between the groups (p=0.0001; Table 3).

**Table 3:** Clinical characteristics of HER2-positive dnMBC patients according to ER and PR expression *Fisher Exact test, vs HER2+RE-RP- group (p=0.04). Values are presented as number (n) and frequency (%). BC=Breast cancer; MT=metastasis; HER2=human epidermic growth factor, receptor 2; ER=estrogen receptor; and PR=progesterone receptor.

Characteristic	All patients (n, %)	HER2+RE+RP- (n, %)	HER2+RE+RP+ (n, %)	HER2+RE-RP- (n, %)	HER2+RE-RP+ (n, %)
Menopausal status					
Menopausic	5 (3.4)	1 (0.7)	2 (1.4)	2 (1.4)	0 (0)
Premenopausal	68 (46.9)	10 (6.9)	26 (17.9)	27 (18.6)	5 (3.4)
Postmenopausal	72 (49.7)	8 (5.5)	31 (21.4)	29 (20)	4 (2.8)
Pregnancies					
>5	18 (12.4)	4 (2.8)	8 (5.5)	4 (2.8)	2 (1.4)
0-2	66 (45.5)	8 (5.5)	26 (17.9)	27 (18.6)	5 (3.4)
3-5	61 (42.1)	7 (4.8)	25 (17.2)	27 (18.6)	2 (1.4)
BC laterality					
Right	77 (53.1)	8 (5.5)	33 (22.8)	31 (21.4)	5 (3.4)
Left	62 (42.8)	10 (6.9)	24 (16.6)	24 (16.6)	4 (2.8)
Both	6 (4.1)	1 (0.7)	2 (1.4)	3 (2.1)	0 (0)
T status					
T1	3 (2.1)	0 (0)	1 (0.7)	2 (1.4)	0 (0)
T2	23 (15.9)	4 (2.8)	10 (6.9)	8 (5.5)	1 (0.7)
T3	25 (17.2)	2 (1.4)	11 (7.6)	10 (6.9)	2 (1.4)
T4	90 (62.1)	13 (9)	36 (24.8)	35 (24.1)	6 (4.1)
Tx	4 (2.8)	0 (0)	1 ( 0.7)	3 (2.1)	0 (0)
N status					
N0	3 (2.1)	0 (0)	1 (0.7)	1 (0.7)	1 (0.7)
N1	19 (13.1)	1 (0.7)	11 (7.6)	5 (3.4)	2 (1.4)
N2	29 (20)	7 (4.8)	12 (8.3)	8 (5.5)	2 (1.4)
N3	93 (64.1)	11 (7.6)	35 (24.1)	43 (29.7)	4 (2.8)
Nx	1 (0.7)	0 (0)	0 (0)	1 (037)	0 (0)
Metastasis sites*					
1	68 (46.9)	6 (4.1)	25 (17.2)	35 (24.1)	2 (1.4)
2	39 (26.9)	4 (2.8)	20 (13.8)	13 (9)	2 (1.4)
3	23 (15.9)	7 (4.8)	8 (5.5)	4 (2.8)	4 (2.8)
4	8 (5.5)	1 (0.7)	3 (2.1)	3 (2.1)	1 (0.7)
5	3 (2.1)	0 (0)	1 (0.7)	2 (1.4)	0 (0)
6	3 (2.1)	0 (0)	2 (1.4)	1 (0.7)	0 (0)
7	1 (0.7)	1 (0.7)	0 (0)	0 (0)	0 (0)
Visceral MT at diagnosis					
Yes	75 (51.7)	7 (4.8)	36 (24.8)	28 (19.3)	4 (2.8)
No	70 (48.3)	12 (8.3)	23 (15.9)	30 (20.7)	5 (3.4)

Pathological status

The histological type of BC was invasive ductal in 124 patients (85.5%), invasive lobular in 15 patients (10.3%), and another type of BC in six (4.1%). SRB scores were, in increasing order, three (five patients, 3.4%), four (five patients, 3.4%), five (six patients, 4.1%), six (26 patients, 17.9%), seven (41 patients, 28.3%), eight (47 patients, 32.4%), and nine (11 patients, 7.6%), with four not reported (2.8%). Severe desmoplasia was reported in 56 patients (38.6%), followed by moderate (39 patients, 26.9%), and mild (29 patients, 20%); desmoplasia was not reported in 21 patients (14.5%). Inflammatory cells in neoplastic tissue were reported in 119 patients (82.1%); whereas lymphovascular and neural invasion were reported in 67 (46.2%) and 20 (13.8%) patients, respectively. Finally, the KI67 biomarker was found to have a higher expression (>45%) in 63 patients (46.4%), followed by percentages in the range of 26-35 (27 patients, 18.6%), 36-45 (22 patients, 15.2%), ≤15 (18 patients, 12.4%) and 16-25 (15 patients, 10.3%). Statistical differences were found only in tumor desmoplasia (p=0.0001; Table 4).

**Table 4:** Pathological characteristics of HER2-positive dnMBC patients according to ER and PR expression * Fisher Exact test, vs HER2+RE-RP- group (p = 0.0001). Values are presented as number (n) and frequency (%). HER2=Human epidermic growth factor, receptor 2; ER=estrogen receptor; and PR=progesterone receptor.

Characteristic	All patients (n, %)	HER2+RE+RP- (n, %)	HER2+RE+RP+ (n, %)	HER2+RE-RP- (n, %)	HER2+RE-RP+ (n, %)
Histological type					
Invasive ductal	124 (85.5)	19 (13.1)	51 (35.2)	46 (31.7)	8 (5.5)
Invasive lobular	15 (10.3)	0 (0)	5 (3.4)	9 (6.2)	1 (0.7)
Other	6 (4.1)	0 (0)	3 (2.1)	3 (2.1)	0 (0)
SBR status					
3	5 (3.4)	0 (0)	2 (1.4)	3 (2.1)	0 (0)
4	5 (3.4)	0 (0)	1 (0.7)	3 (2.1)	1 (0.7)
5	6 (4.1)	0 (0)	3 (2.1)	3 (2.1)	0 (0)
6	26 (17.9)	6 (4.1)	11 (7.6)	9 (6.2)	0 (0)
7	41 (28.3)	4 (2.8)	20 (13.8)	16 (11)	1 (0.7)
8	47 (32.4)	6 (4.1)	18 (12.4)	18 (12.4)	5 (3.4)
9	11 (7.6)	3 (2.1)	3 (2.1)	3 (2.1)	2 (1.4)
Not reported	4 (2.8)	0 (0)	1 (0.7)	3 (2.1)	0 (0)
Desmoplasia*					
Mild	29 (20)	5 (3.4)	14 (9.7)	10 (6.9)	0 (0)
Moderate	39 (26.9)	9 (6.2)	13 (9)	16 (11)	1 (0.7)
Severe	56 (38.6)	5 (3.4)	22 (15.2)	22 (15.2)	7 (4.8)
Not reported	21 (14.5)	0 (0)	10 (6.9)	10 (6.9)	1 (0.7)
Presence of inflammatory cells					
No	6 (4.1)	1 (0.7)	1 (0.7)	4 (2.8)	0 (0)
Not reported	20 (13.8)	0 (0)	8 (5.5)	11 (7.6)	1 (0.7)
Yes	119 (82.1)	18 (12.4)	50 (34.5)	43 (29.7)	8 (5.5)
Lymphovascular invasion					
No	66 (45.5)	9 (6.2)	20 (13.8)	32 (22.1)	5 (3.4)
Not reported	12 (8.3)	1 (0.7)	5 (3.4)	5 (3.4)	1 (0.7)
Yes	67 (46.2)	9 (6.2)	34 (23.4)	21 (14.5)	3 (2.1)
Neural invasion					
No	102 (70.3)	14 (9.7)	39 (26.9)	42 (29)	7 (4.8)
Not reported	23 (15.9)	2 (1.4)	11 (7.6)	9 (6.2)	1 (0.7)
Yes	20 (13.8)	3 (2.1)	9 (6.2)	7 (4.8)	1 (0.7)
KI67					
>45	63 (43.4)	10 (6.9)	20 (13.8)	28 (19.3)	5 (3.4)
≤15	18 (12.4)	3 (2.1)	10 (6.9)	4 (2.8)	1 (0.7)
16-25	15 (10.3)	1 (0.7)	9 (6.2)	5 (3.4)	0 (0)
26-35	27 (18.6)	3 (2.1)	12 (8.3)	11 (7.6)	1 (0.7)
36-45	22 (15.2)	2 (1.4)	8 (5.5)	10 (6.9)	2 (1.4)

Receptor status

The expression of estrogen receptor was reported as a range with a value of 201-300 (12 patients, 8.3%) followed by 101-200 in 42 patients (29%), 51-100 (six patients, 4.1%) and <50 in 18 patients (12.4%). In the same way, progesterone receptors in tumoral tissue were reported as ranges; a value of 201-300 was found in six patients (4.1%), followed by 101-200 in 21 patients (14.5%) , 51-100 in 12 patients (8.3%) and <50 in 29 patients (20%). HER2 receptor status was positive in 137 patients (94.5%) and indeterminate in eight patients (5.5%); for this group, a fluorescence in situ hybridization (FISH) analysis was carried out to confirm the overexpression of HER2 (Table 5). Statistical differences were found only in estrogen and progesterone receptors in tumors between groups (p=0.0001).

**Table 5:** Receptor expression in HER2-positive dnMBC patients Fisher Exact test, * vs HER2+RE+RP+ group (p=0.0001); # vs HER2+RE-RP+ group (p=0.0001). Values are presented as number (n) and frequency (%). HER2=Human epidermic growth factor, receptor 2; ER=estrogen receptor; PR=progesterone receptor.

Receptor Type	All patients (n, %)	HER2+RE+RP- (n, %)	HER2+RE+RP+ (n, %)	HER2+RE-RP- (n, %)	HER2+RE-RP+ (n, %)
Estrogens*					
<50	18 (12.4)	10 (6.9)	8 (5.5)	0 (0)	0 (0)
101–200	42 (29)	8 (5.5)	34 (23.4)	0 (0)	0 (0)
201–300	12 (8.3)	1 (0.7)	11 (7.6)	0 (0)	0 (0)
51–100	6 (4.1)	0 (0)	6 (4.1)	0 (0)	0 (0)
Progesterone^#^					
<50	29 (20)	0 (0)	22 (15.2)	0 (0)	7 (4.8)
101-200	21 (14.5)	0 (0)	21 (14.5)	0 (0)	0 (0)
201-300	6 (4.1)	0 (0)	6 (4.1)	0 (0)	0 (0)
51-100	12 (8.3)	0 (0)	10 (6.9)	0 (0)	2 (1.4)
HER2					
2+	8 (5.5)	1 (0.7)	6 (4.1)	1 (0.7)	0 (0)
3+	137 (94.5)	18 (12.4)	53 (36.6)	57 (39.3)	9 (62)

Treatment

Palliative mastectomy was performed in 22 (15.2%) patients; with regard to radiotherapy, 87 (60%) patients received radiation on the breast, followed 51 patients on the brain (35.2%) and 23 patients on the backbone (15.9%). At least one line of treatment was offered to all patients, up to 10 lines in one patient (Table 6). Statistical differences was found only in surgery (palliative mastectomy) between the analyzed groups (p=0.001).

**Table 6:** Provided treatment for HER2-positive dnMBC patients according to ER and PR expression Fisher's exact test, *vs HER2+RE-RP- group (p=0.001). Values are presented as number (n) and frequency (%). HER2=human epidermic growth factor, receptor 2; ER=estrogen receptor; PR=progesterone receptor.

Characteristic	All Patients (n, %)	HER2+ER+PR- (n, %)	HER2+RE+RP+ (n, %)	HER2+RE-RP- (n, %)	HER2+RE-RP+ (n, %)
Surgery*					
Palliative mastectomy	22 (15.2)	2 (10.5)	6 (10.2)	13 (22.4)	1 (11.1)
Radiotherapy site					
Breast	87 (60)	12 (63.2)	39 (66.1)	31 (53.4)	5 (55.6)
Vertebral column	23 (15.9)	1 (5.3)	11 (18.6)	6 (10.3)	5 (55.6)
Brain	51 (35.2)	4 (21.1)	16 (27.1)	26 (44.8)	5 (55.6)
Other	15 (10.3)	4 (21.1)	6 (10.2)	4 (6.9)	1 (11.1)
Lines of treatment					
1L	145 (100)	19 (100)	59 (100)	58 (100)	9 (100)
2L	127 (87.6)	16 (84.2)	54 (91.5)	50 (86.2)	7 (77.8)
3L	93 (64.1)	12 (63.2)	44 (74.6)	31 (53.4)	6 (66.7)
4L	59 (40.7)	8 (42.1)	27 (45.8)	20 (34.5)	4 (44.4)
5L	44 (30.3)	7 (36.8)	23 (39)	10 (17.2)	4 (44.4)
6L	32 (22.1)	4 (21.1)	19 (32.2)	6 (10.3)	3 (33.3)
7L	21 (14.5)	4 (21.1)	9 (15.3)	5 (8.6)	3 (33.3)
8L	11 (7.6)	3 (15.8)	4 (6.8)	2 (3.4)	2 (22.2)
9L	5 (3.4)	2 (10.5)	2 (3.4)	0 (0)	1 (11.1)
10L	1 (0.7)	0 (0)	1 (1.7)	0 (0)	0 (0)

Survival

**Figure 2:**
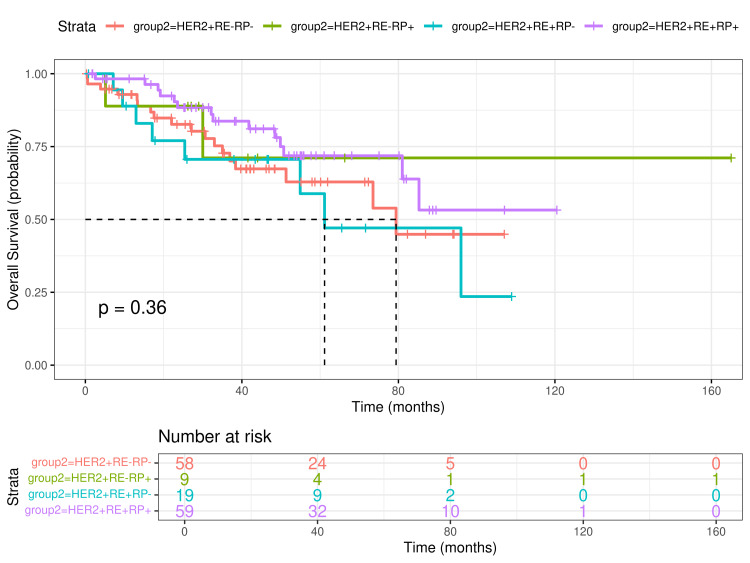
Overall survival (OS) in patients with HER2-positive dnMBC according to ER and PR expression The upper panel shows survival rates for the different groups. Data was analyzed with a log-rank test. The lower panel shows the number of patients at risk by time and by ER and PR expression. dnMBC=De novo metastatic breast cancer; HER2=Human epidermic growth factor, receptor 2; ER=estrogen receptor; PR=progesterone receptor

**Figure 3:**
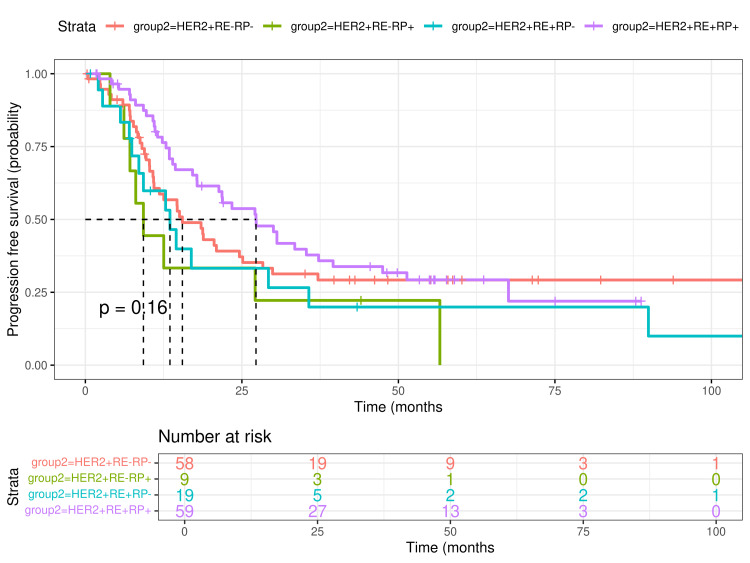
Progress-free survival (PFS) in patients with HER2-positive dnMBC according to ER and PR expression The upper panel shows survival rates for the different groups. Data was analyzed with a log-rank test. The lower panel shows the number of patients at risk by time and by ER and PR expression. dnMBC=De novo metastatic breast cancer; HER2=Human epidermic growth factor, receptor 2; ER=estrogen receptor; PR=progesterone receptor

OS was 96 months (median; 95% CI: 79 to not estimable), whereas median PFS was 18.7 months (95% CI: 14.5-27.3). The median of estimated OS was 79.4 months (95% CI: 51.3 to not estimable) for the HER2+ER-PR- group and 61.1 months (95% CI: 54.9 to not estimable) for the HER2+ER+PR+ group. The medians for the HER2+ER-PR+ and HER2+ER+PR- groups were not reached. The median of PFS was 15.5 months (95% CI: 10.9-28.3), 9.27 months (95% CI: 7.12 to not estimable), 13.51 months (95% CI: 8.54 to not estimable), and 27.2 months (95% CI: 17.85-39.6), for the HER2+ER-PR-, HER2+ER-PR+, HER2+ER+PR-, and HER2+ER+PR+ groups, respectively. The survival time rate for one through five years was 94% (95% CI: 90-98), 84% (95% CI: 78-91), 77% (95% CI: 69-85), 73% (95% CI: 66-82), and 66% (95% CI: 57-76). No statistical differences were found in the log-rank test for any group in OS or PFS (Figures 2, 3).

A multivariate Cox regression was performed to determine the influence of social variables on the survival rates of dnMBC, but no factors associated with the outcome were found in the social characteristics when analyzed under the model specifications (Table 7).

**Table 7:** Multivariate Cox regression results in patients with HER2-positive dnMBC HER2=Human epidermic growth factor, receptor 2; ER=estrogen receptor; PR=progesterone receptor; HR=hazard ratio; CI=confidence interval; NE=not estimable.

Variable / Group	HR	Lower 95% CI	Upper 95% CI	p value
Receptor status (vs HER2+RE-RP-)				
HER2+RE+RP+	0.560	0.242	1.299	0.1768
HER2+RE+RP-	1.311	0.496	3.465	0.5855
HER2+RE-RP+	0.704	0.156	3.179	0.6477
Age (vs <50 years)				
≥ 51years	0.708	0.313	1.598	0.4056
Marital status (vs widowed)				
Married	0.510	0.158	1.654	0.2624
Divorced	0.635	0.149	2.708	0.5393
Single	0.465	0.127	1.704	0.2478
Common-law	0.546	0.124	2.405	0.4240
Insurance status (vs not insured)				
Insurance	0.000	0.000	NE	0.9945
Geographical origin (vs Southeast)				
Center	1.824	0.604	5.511	0.2868
West	0.000	0.000	NE	0.9957
Educational level (vs technical)				
Illiterate	2.281	0.199	26.154	0.5076
Bachelor’s	0.504	0.023	10.834	0.6615
High school	1.299	0.124	13.547	0.8271
Elementary	1.073	0.122	9.443	0.9495
Secondary	1.370	0.150	12.509	0.7804
Occupation (vs technical)				
Housewife	0.233	0.000	NE	0.9996
Unemployed	0.335	0.000	NE	0.9997
Retired	0.516	0.000	NE	0.9999
Non professional	0.194	0.000	NE	0.9996
Professional	0.112	0.000	NE	0.9995

## Discussion

The present study described the social, clinical, and pathological characteristics and treatment patterns as well as exploratory survival outcomes in patients with HER2-positive dnMBC for the Mexican population in a reference center for cancer care. Breast cancer is the most frequent form of cancer in women and the leading cause of mortality; in 2020, the incidence was 39.5 and mortality was 9.9 per 100,000 women [7]. We found an overall incidence of dnMBC of 39% in HER2-positive MBC patients; these results are consistent with previous research in which the incidence was 28.8%-40.2% [8-12].

According to the data, the median follow-up time was 42 months (with a range of 27 days to 166 months) with no differences in hormonal receptor status between subgroups; this time is similar to that reported by Han-Fang [8] (42.8 months) and by Smith [13] (90 months in patients with durable response). This finding could be related to treatment patterns in HER2-positive dnMBC as reported by Smith [13]. Trastuzumab-Pertuzumab-Carboplatin-Docetaxel (TCHP)-based treatments are associated with prolonged responses, which could support our findings.

Regarding social and demographic characteristics, the results are consistent with previous research; for example, we found age ranges comparable to similar studies [13-15]. Conversely, our study reports on social variables in HER2-positive dnMBC. Married women, housewives, uninsured women, and women with elementary education were most frequently patients with BC; in a review, Coughlin [16] stated that social determinants such as poverty, lack of education, lack of social support, and social isolation are associated with late-stage BC diagnosis, reflecting inadequate access to medical care, consistent with our own findings. Finally, most of the patients came from center of Mexico, which can be explained by the fact that the National Cancer Institute is the reference center for highly specialized cancer care in the country for patients without medical insurance.

Reproductive factors such as early menarche, late menopause, late age at first pregnancy, and low parity can increase the risk of breast cancer [17]. For HER2-positive dnMBC patients, this is the first study in which menarche age and pregnancies are reported in a descriptive way; the menopausal status data were consistent with other studies with the same population in which postmenopausal women were the most frequent status reported, ranging from 69.9% to 77.3% [18,19].

The reported clinical burden of disease was above those reported by other authors. There was a high frequency of T4 status and N3 node status. Previously, the tumor burden had not been reported for this population; however, other comparative studies between dnMBC and recurrent metastatic breast cancer (rMBC) have reported the TN status. So, Almasri and colleagues [10] found that T2 status was more frequent than other T statuses; also, 74% of patients were positive for lymph node status. Conversely, File and colleagues [20] reported a proportion of 51.7% for T3/T4 status, and 16.1% of patients had positive nodes. The number of sites with metastasis and presence of visceral disease coincided with previous studies in which few sites were found in up to 60% of the patients [14,15,18,21,22]. As reported by others, this burden of disease is characteristic for metastatic breast cancer [23] and our observations corresponded with those findings.

Several pathological features have not been reported previously for HER2-positive dnMBC; the present study has documented characteristics such as inflammatory cells and the presence of desmoplasia in tumor tissue as well as lymphovascular and neural infiltration by BC cells. However, for MBC (both de novo and recurrent), some studies [9,10,18-20] have documented invasive ductal carcinoma, high-grade tumors, and elevated Ki67 tumor expression as reported in the present study. All studies discussed tumoral characteristics; these have been extensively reviewed [24,25] and match our findings.

One point of interest was to classify HER2-positive dnMBC in accordance with ER and PR expression; in this study, 53% and 46% of dnMBC cases had any level of estrogen or progesterone expression, respectively, and these values are in concordance with other reports with similar populations [13,15,18]. The findings regarding the strength of ER or PR positivity could be predictive factors for survival, progression, or treatment response, as reported by Morgan [26]; however, the existing data in HER2-positive dnMBC is limited and too heterogeneous to make inferences about its usefulness in disease behavior.

We reported a median OS of 96 months and a median PFS of 18.7 months for all patients; these findings were higher than others previously published; Yardley et al. [21] reported an OS of 41.7 months (95% CI: 36.1-47.2) and a PFS 12.1 of months (95% CI: 11.4-13.5) in the context of dnMBC. In the same way, Tripathy et al. [14] reported a PFS of 17.7 months (95% CI: 16.0-19.7); other reports in the same population [18] showed OS and PFS of 55.9, and 14.4 months, respectively. This difference in OS could be due to treatment patterns, as reported by Lambertini et al. [18]; for this study, we noted that patients were treated with several modalities, which could be the main reason for this difference. To clarify this, a subgroup analysis was done; surprisingly, for OS, the HER2+ER-PR+ was 79.4 months with HR of 0.70 (95% CI, 0.15-3.17) and, 61.1 months for HER2+ER+PR+ with a hazard ratio (HR) of 0.56 (95% CI: 0.24-1.29) without statistical significance. As an exploratory analysis, several factors and their effect on survival was inconclusive and the study does not clearly show if these factors are responsible for the survival and to what extent; so, more research is needed to clarify this finding.

Our study showed a survival rate of 94% from year one to 66% in year five; several publications have documented the survival rates of dnMBC in comparison with recurrent metastatic BC (rMBC); in one, the overall survival at year one was 84% and 28.9% in year five [20]. Another study [10] showed a 32.6% survival rate at year five; for the mentioned studies, the cohorts included HER2-positive and negative cases, which might cause the results to be underestimated. Conversely, Wong and colleagues [15] reported survival rates of 98% in years five and 10 in HER2-positive patients with no evidence of disease after treatment with anti-HER2 therapy; this finding supports the idea that receptor status (HER2, estrogen, or progesterone) is directly related to survival outcomes. The present study reports treatment patterns, but future research is needed to clarify this statement.

Despite our interest in identifying the influence of social variables on the survival for HER2-positive dnMBC patients, multivariate analysis did not show any factors associated with survival. However, this study reports variables such as marital status, geographical origin, level of education, insurance status, and occupation. So, the lack of any difference could be associated with tumor biology instead of social and economic variables, as reported by Soares et al. [27]. More research is needed to associate social variables with clinical outcomes in HER2-positive dnMBC.

Some limitations were observed; as a retrospective study, missing or incomplete data could affect the interpretation of results, which should be treated with some caution. This is only a limited study, and the full potential of the results should be considered in controlled conditions as randomized clinical trial. Closer examinations in survival results showed some anomalies, for example the high lost-to-follow up rate led us to question its suitability for real results; so, our results should be considered as exploratory only. The data was collected from a single institution specializing in cancer over 10 years, which could increase the robustness of findings, but more research should be done to clarify the social, clinical, and pathological relationships of HER2-positive dnMBC.

## Conclusions

Breast cancer is a leading cause of mortality and morbidity worldwide; in any clinical stage, it represents a high burden of disease in the healthcare system because medical care is sometimes associated with higher resource utilization than other diseases. Recently, differences in survival between de novo and recurrent metastatic breast cancer have been reported, but molecular receptor expression has not been extensively characterized and correlated with survival outcomes. This study provides descriptive results of clinical, sociodemographic, treatment patterns, and pathological characteristics according with molecular receptor status. The reported survival outcomes should be interpreted as exploratory due to high lost-to-follow up rate, the main limitation of this retrospective study.

## References

[1] Giaquinto AN, Sung H, Newman LA (2024). Breast cancer statistics 2024. CA Cancer J Clin.

[2] Lei S, Zheng R, Zhang S (2021). Global patterns of breast cancer incidence and mortality: a population-based cancer registry data analysis from 2000 to 2020. Cancer Commun (Lond).

[3] Daily K, Douglas E, Romitti PA, Thomas A (2021). Epidemiology of de novo metastatic breast cancer. Clin Breast Cancer.

[4] Boscolo Bielo L, Trapani D, Nicolò E (2024). The evolving landscape of metastatic HER2-positive, hormone receptor-positive breast cancer. Cancer Treat Rev.

[5] El Masri J, Phadke S (2022). Breast cancer epidemiology and contemporary breast cancer care: a review of the literature and clinical applications. Clin Obstet Gynecol.

[6] Swain SM, Shastry M, Hamilton E (2023). Targeting HER2-positive breast cancer: advances and future directions. Nat Rev Drug Discov.

[7] SMEO: Consenso Mexicano sobre el diagnóstico y tratamiento del cáncer mamario (2023). SMEO: Consenso Mexicano sobre el diagnóstico y tratamiento del cáncer mamario. http://consensocancermamario.com/.

[8] Cheng HF, Tsai YF, Huang CC (2022). Clinical outcomes and metastatic behavior between de novo versus recurrent HER2-positive metastatic breast cancer: a 17-year single-institution cohort study at Taipei Veterans General Hospital. J Chin Med Assoc.

[9] Zheng A, Guo BL, Zhang JG, Jin F (2021). Clinical information and management status of de novo stage IV breast cancer patients: a Chinese multicenter investigation (CSBrS-002). Chin Med J (Engl).

[10] Almasri H, Erjan A, Abudawaba H, Ashouri K, Mheid S, Alnsour A, Abdel-Razeq H (2022). Clinical characteristics and survival outcomes of patients with de novo metastatic breast cancer. Breast Cancer (Dove Med Press).

[11] Zhao W, Wu L, Zhao A (2020). A nomogram for predicting survival in patients with de novo metastatic breast cancer: a population-based study. BMC Cancer.

[12] Zhao J, Bian S, Di X, Xiao C (2023). A nomogram and risk classification system predicting the prognosis of patients with de novo metastatic breast cancer undergoing immediate breast reconstruction: a surveillance, epidemiology, and end results population-based study. Curr Oncol.

[13] Smith CE, Marcom PK, Mitri Z, Ko NY (2022). Predictors of long-term durable response in de novo HER2-positive metastatic breast cancer and the real-world treatment experience at two institutions. Breast Cancer Res Treat.

[14] Tripathy D, Brufsky A, Cobleigh M (2020). De novo versus recurrent HER2-positive metastatic breast cancer: patient characteristics, treatment, and survival from the SystHERs registry. Oncologist.

[15] Wong Y, Raghavendra AS, Hatzis C (2019). Long-term survival of de novo stage IV human epidermal growth receptor 2 (HER2) positive breast cancers treated with HER2-targeted therapy. Oncologist.

[16] Coughlin SS (2019). Social determinants of breast cancer risk, stage, and survival. Breast Cancer Res Treat.

[17] Sun YS, Zhao Z, Yang ZN (2017). Risk factors and preventions of breast cancer. Int J Biol Sci.

[18] Lambertini M, Ferreira AR, Di Meglio A (2017). Patterns of care and clinical outcomes of HER2-positive metastatic breast cancer patients with newly diagnosed stage IV or recurrent disease undergoing first-line trastuzumab-based therapy: a multicenter retrospective cohort study. Clin Breast Cancer.

[19] Kotoula V, Tsakiri K, Koliou GA (2019). Relapsed and de novo metastatic HER2-positive breast cancer treated with trastuzumab: tumor genotypes and clinical measures associated with patient outcome. Clin Breast Cancer.

[20] File DM, Pascual T, Deal AM, Wheless A, Perou CM, Claire Dees E, Carey LA (2022). Clinical subtype, treatment response, and survival in de novo and recurrent metastatic breast cancer. Breast Cancer Res Treat.

[21] Yardley DA, Kaufman PA, Brufsky A (2014). Treatment patterns and clinical outcomes for patients with de novo versus recurrent HER2-positive metastatic breast cancer. Breast Cancer Res Treat.

[22] McAndrew EN, Graham J, Dufault B, Desautels DN, Kim CA (2024). Long-term survival among patients with de novo human epidermal growth receptor 2-positive metastatic breast cancer in Manitoba. Am J Clin Oncol.

[23] Yang SX, Hewitt SM, Yu J (2022). Locoregional tumor burden and risk of mortality in metastatic breast cancer. NPJ Precis Oncol.

[24] Weigelt B, Geyer FC, Reis-Filho JS (2010). Histological types of breast cancer: how special are they?. Mol Oncol.

[25] Seltzer S, Corrigan M, O'Reilly S (2020). The clinicomolecular landscape of de novo versus relapsed stage IV metastatic breast cancer. Exp Mol Pathol.

[26] Morgan DA, Refalo NA, Cheung KL (2011). Strength of ER-positivity in relation to survival in ER-positive breast cancer treated by adjuvant tamoxifen as sole systemic therapy. Breast.

[27] Soares LR, Freitas-Junior R, Curado MP, Paulinelli RR, Martins E, Oliveira JC (2020). Low overall survival in women with de novo metastatic breast cancer: does this reflect tumor biology or a lack of access to health care?. JCO Glob Oncol.

